# A Case Report on Multisystem Inflammatory Syndrome After COVID-19 Infection in a 12-Year-Old Child

**DOI:** 10.7759/cureus.29061

**Published:** 2022-09-11

**Authors:** Piyush U Kathane, Keta Vagha

**Affiliations:** 1 Community Medicine, Jawaharlal Nehru Medical College, Datta Meghe Institute of Medical Sciences, Wardha, IND; 2 Pediatrics, Acharya Vinoba Bhave Rural Hospital, Datta Meghe Institute of Medical Sciences, Wardha, IND

**Keywords:** pediatrics, covid-19 virus, multisystem organ failure, children, liver failure, liver inflammation, sars-cov-2 virus, covid-19 infection, inflammation, misc

## Abstract

Several new diagnostic and management challenges have evolved as a result of limited knowledge about the incidence of multisystem inflammatory syndrome in children (MISC) and its natural history which is caused by coronavirus disease 2019 (COVID-19) infection. There are no data on the long-term outcomes of the illness and MISC. A 12-year-old female child with no significant history was rushed to a emergency room with complaints of rashes all over her body for the past seven days, fever for the past five days, and altered sensorium for the last one day. The child tested negative for COVID-19 at first and then was found to be antibody positive for COVID-19. The child showed symptoms of the multisystem inflammatory syndrome, which involved the main alteration in liver functioning. This case is suggestive of how the COVID-19 virus causes alteration in liver functioning and its inflammation, including inflammation of other organs, which is called MISC.

## Introduction

In December 2019, a unique severe acute respiratory syndrome coronavirus 2 (SARS-CoV-2) outbreak occurred with various symptoms, mainly respiratory and liver infections. The World Health Organization (WHO) declared coronavirus disease 2019 (COVID-19) a pandemic on March 11, 2020, but immunization is now available for the SARS-CoV-2 illness [1]. In relation to the rapidly spreading COVID-19 pandemic, the majority of observations showed severe clinical signs and symptoms in children. In some cases, it can cause single or multiorgan inflammation and single or multiorgan failure and systemic failure in adults as well as children [2]. Multisystem inflammatory syndrome in children (MISC) is a severe condition that appears to be linked to COVID-19. In children who develop MISC, there is intense inflammation in some organs and tissues such as the lungs, heart, blood vessels, kidneys, brain, skin, eyes, and digestive system. According to studies, the main target of MISC is considered to be the liver [3]. Out of the total patients infected with COVID-19 worldwide, up to 60% of patients infected with COVID-19 showed inflammation in the liver. Patients showed elevation in liver enzymes, and these elevations are seen more in serious patients. Obesity is the most frequent and common comorbidity found in children who are hospitalized with COVID-19 infection and is highly associated with hepatitis [4]. There is fever, accompanied many times by severe pain in the abdominal region, progressive respiratory failure, and myocardial dysfunction with varying degrees of cardiogenic shock and circulatory failure in patients with MISC [5]. These symptoms can get more severe to the point that there may be a need for organ replacement, including extracorporeal membrane oxygenation and hemodialysis, or even mechanical ventilation. Therefore, the mortality of this illness is severely different from the course previously reported for COVID-19 infection in most pediatric patients [6].

## Case presentation

A 12-year-old female was brought to emergency room with complaints of rashes all over her body for the last seven days, fever for the last five days, and altered sensorium from the past one day. The patient was brought to the emergency room in shock. In the emergency room, she had tachycardia, pulses were feeble, saturation was 89% on room air, and blood pressure was 60/40 mmHg. The mother reported that the patient was asymptomatic seven days prior. Then she started complaining of rashes all over her body which were erythematous, non-pruritic, and initially appeared on the chest and trunk but progressed to involve the entire body. She was given traditional treatment at home (applied turmeric, bath with hot water and neem leaves, and turmeric-infused milk and tea). Two days later she developed high-grade fever, insidious in onset, progressive in nature, not associated with chills and rigors, with no history of contact with COVID-19 patients. The fever was relieved by taking medication (paracetamol 500 mg). One day before the presentation in emergency room, she had altered sensorium with difficulty talking. On examination, the liver was palpable at 4 cm below the costal margin. The chest was bilaterally clear with no added sounds. The patient suddenly went into septic shock. A rapid antigen test for COVID-19 was done, and she was admitted to the pediatric intensive care unit (PICU) after testing negative. After admission, she was kept nil-by-mouth and was started on oxygen by nasal prongs, intravenous (IV) fluids, noradrenaline, and adrenaline drips. Venous blood gas analysis was suggestive of compensated metabolic acidosis. One unit of packed red blood cells was transfused after blood investigations. On day one, a blood culture was sent, and the results of the complete blood count (CBC) are presented in Table 1 [7].

**Table 1:** Complete blood count findings of the patient and the normal range.

	Findings	Normal range
Hemoglobin	10.7 g/dL	Malea: 13.2–16.6 g/dL; females: 11.6–15 g/dL
Total leucocyte count	20,300/mm^3^	4,000–11,000/mm^3^
Platelets	100,000/µL	150,000–450,000/µL
Serum alanine transaminase	158 U/L	4–36 U/L
Serum aspartate transaminase	149 U/L	8–33 U/L
Serum bilirubin	1.8 mg/dL	1 mg/dL for under 18
Conjugated bilirubin	1.1 mg/dL	less than 0.3 mg/dL
Serum albumin	2.6 (L) g/dL	3.4–5.4 g/dL
Serum urea	82 (H) mg/dL	6–24 mg/dL
Serum creatinine	1.4 mg/dl	0.74–1.35 mg/dL
Serum potassium	5.5 mEq/L	3.5–5.5 mEq/L
Prothrombin time	15 seconds	11–13.5 seconds
International normalization ratio	1.3	1.0

Chest X-ray was suggestive of bilateral infiltrates with increased bronchoalveolar margins. The patient was started on potassium-free IV fluids, meropenem, and vancomycin.

On the second day, the patient had continuous episodes of high-grade fever, nasal flaring, and tachypnea. She was upgraded to oxygen by constant positive airway pressure. She was started on methylprednisolone, enoxaparin, and IV immunoglobulins after testing antibody positive for COVID-19. She was then upgraded to oxygen by continuous positive airway pressure. A two-dimensional echo was done which was suggestive of an ejection fraction of 50%, mild tricuspid regurgitation, and mild pulmonary arterial hypertension. Milrinone was started in continuous drip. Nonstructural protein 1 antigen and antibodies for dengue were negative. The patient also tested negative for hepatitis B surface antigen and hepatitis C virus. Subsequent CBCs were suggestive of an increased total leukocyte count of 24,000 mm^3^ (normal: 4,000-11,000/mm^3^). On examination, bilateral coarse crepitations were present. A repeat chest X-ray was suggestive of increased involvement of lungs with increased infiltration. Colistin was added. An inflammatory panel was sent, and the results are presented in Table 2 [8].

**Table 2:** Blood test findings of the patient and the normal range.

	Findings	Normal range
Erythrocyte sedimentation rate	24 mm/hour	Males: 0–22 mm/hour; females: 0–29 mm/hour
C-reactive protein	20 mg/L	Less than 10 mg/L
Troponin I	180 ng/mL	0–0.04 ng/mL
Serum ferritin	255 µg/L	Males: 24–336 µg/L; females: 11–307 µg/L
Lactate	2.7 mmol/L	2.3 mmol/L
D-dimer	8 µg/mL	0.4 µg/mL
lactate dehydrogenase	565 (H) IU/L	105–333 IU/L

Considering the diagnosis of MISC, antibodies for COVID-19 were also sent. She tested positive for the same and was started on IV immunoglobulins, enoxaparin, and methylprednisolone.

Outcomes and follow-up

Gradually over a period of 14 days, the patient started to show signs of improvement. Irritability was decreased, sensorium was improved, and repeat serum alanine transaminase, serum aspartate transaminase, serum urea, and serum creatinine decreased. Repeat inflammatory markers were also in a reducing trend. Respiratory distress decreased over time, and she was subsequently weaned off from supplemental oxygen. She was allowed an oral diet. Antibiotics were given and omitted later. The patient was accepting and tolerating feeds very well. The patient was also maintaining oxygen saturation on room air and was later discharged from the hospital with advice to follow-up in the outpatient department after 15 days.

## Discussion

**Figure 1:**
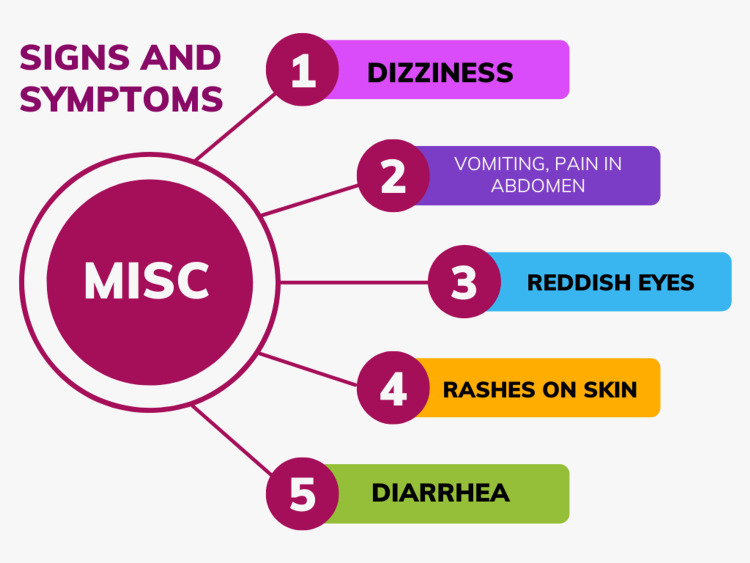
Signs and symptoms of MISC. MISC: multisystem inflammatory syndrome in children

The signs and symptoms of MISC are shown in Figure 1 [9].

COVID-19 is a life-threatening disease for children which may cause systemic diseases and can involve other organs such as the liver, lungs, and the entire respiratory system due to angiotensin-converting enzyme-2 (ACE2) by the distribution of primary viral entry receptor. It is found that even after the recovery (real-time reverse transcription polymerase chain reaction negative) of patients from COVID-19, the symptoms persist or new symptoms develop, and investigations show negative results for RTPCR and rapid antigen test [10]. However, antibody testing can be done to detect the COVID-19 virus to confirm if they have been affected by the virus in the past and have recovered, even if asymptomatic. Liver damage in COVID-19 is defined as damage to the liver or functioning of the liver caused in the entire course of infection or treatment with or without previous infection or damage to the liver or functioning of the liver [11]. Liver infection and liver damage are common in MISC. Moderate steatosis, lobular and portal inflammation, and elevated plasma alanine transaminase (ALT) and aspartate aminotransferase (AST) are all symptoms of COVID-19, which are linked to hepatocellular damage. Patients with higher levels of serum ALT have higher levels of C-reactive protein (CRP) synthesized by the liver. D-dimer, ferritin, and interleukin-6 (IL-6) monocytes, macrophages, and T cells create IL-6 in response to immune system activation, which encourages the generation of CRP and elevated IL-6 levels, which are linked to liver injury in COVID-19 [12]. The virus spike protein binds to ACE2 to gain access into the cell, and infection requires the transmembrane serine protease 2 and paired essential amino acid cleaving enzyme [13]. Consequently, the presence of these receptors serves as an early indicator of permissive hepatic cells [14]. The pathophysiology of COVID-19 and MISC liver involvement is still unknown. However, it is considered that hepatocellular infection is induced by the direct cytopathic effect of SARS-CoV-2 or an immune-mediated response to inflammatory damage [15]. In addition, there might be involvement of hypoxic shock or shock-related circulatory compromise, endothelial dysfunction, microthrombi formation, and drug-induced liver injury [16].

Summary

A 12-year-old female child was brought with complaints of rashes all over the body, fever, and confusion. At first, she was given home treatment for the same, but the symptoms persisted. After bringing her to the hospital, doctors took her to the emergency room. The child was COVID-19 negative on investigation, but after antibody testing, she was found to be positive for COVID-19. Subsequently, after investigations for liver function and examinations of the liver and other organs, the treatment was given accordingly. The child is suffering from liver damage and altered liver function caused by post-COVID-19 infection, which is suggestive of MISC, which is caused by COVID-19 infection.

## Conclusions

We observed MISC in a 12-year-old female with COVID-19 infection, which showed cardiac dysfunction, respiratory failure, and liver injury. COVID-19 associated with MISC requires more investigations and research to improve patient care. A thorough follow-up and case study are necessary to help clinicians identify the spectrum of symptoms of MISC, which is needed for timely identification and appropriate treatment of the patients.
